# Fracture rates by medication type in attention-deficit/hyperactive disorder

**DOI:** 10.3389/fsurg.2023.973266

**Published:** 2023-02-15

**Authors:** Jason P. Sidrak, Syler R. Blaakman, Elijah W. Hale

**Affiliations:** ^1^ School of Medicine, University of Colorado, Aurora, CO, United States; ^2^ University of Rochester, Rochester, NY, United States

**Keywords:** ADHD (Attention deficit and hyperactivity disorder), fractures – bone, bone densities, pharmacology, orthopedic

## Abstract

**Background:**

ADHD is a condition with extensively researched increased risks of psychiatric disorders, traumatic injury, impulsivity, and delayed response times.

**Objectives:**

To analyze the incidences of fractures in patients with ADHD on various medication regimens.

**Methods:**

Using the TriNetX database, we created seven patient cohorts, all of age under 25, based on medication types commonly used for ADHD. The cohorts we created were: no medication use, exclusive use of a -phenidate class stimulant, exclusive use of an amphetamine class stimulant, nonexclusive use of formations of either stimulant, exclusive use of non-stimulant medications approved for ADHD, nonexclusive use, and no medications. We then examined rates while controlling for age, sex, race, and ethnicity.

**Results:**

The comparison of ADHD to neurotypical individuals revealed an increased risk for all fracture types. For the controlled analysis, all but one cohort had significant differences in each fracture type compared to the baseline cohort of ADHD patients without any medication use. Patients in the “phenidate” cohort had an insignificant difference in risk of lower limb fractures. Patients in the “any medication,” “-etamine,” “stimulant,” and “not ADHD” groups all had significant decreased risks for all fracture types, with confidence intervals often overlapping between treatment modalities.

**Conclusions:**

As patients experiment with different medication regimens, providers should be aware of the difference in risk of fracture by medication type. Our results highlight the need for continued research to better discern appropriate medication regimens with the goal of improving overall risk reduction and producing better outcomes for individuals with ADHD.

## Introduction

Attention-Deficit/Hyperactivity Disorder (ADHD) is one of the most common developmental disorders, affecting up to 10% of the population (1). ADHD is a condition with extensively researched increased risks of psychiatric disorders, traumatic injury, impulsivity, and delayed response times (1). The heterogenous nature of ADHD necessitates scientific inquiry and discovery into all associated co-morbidities, environmental and social impacts, and other exacerbating factors to ensure treatment is all encompassing.

A significant yet often overlooked ADHD association is difficulty in motor coordination and tasks requiring complex coordinated movements (2). These motor difficulties are often interpreted socially as the individual being clumsy or incorrectly attributed to poor focus on surroundings/tasks due to attention-deficit and hyperactivity. They are also postulated to contribute to the statistically significant risk of accidental traumatic injuries seen in children with ADHD (3). A nationwide matched study found that the incidence of fractures in people with ADHD was 1.4 times greater than the rates seen in those without ADHD (4). An earlier study controlled for confounding factors such as socioeconomic status, and still arrived at an increased risk of 1.32 (5). Importantly, the earlier study by Chou et al. also controlled for certain conditions, such as developmental coordination disorder, that could have caused an increased fracture risk on their own (5).

In response to these well-studied increased injury risks, some recent studies have attempted to investigate whether stimulant treatment leads to protective musculoskeletal benefits. While investigating stimulants is a worthwhile endeavor, there are no studies comparing the impact of all available ADHD medications on musculoskeletal injuries. The objective of our study is to analyze the incidences of fractures in patients with ADHD on various medication regimens.

## Methods

We performed a retrospective study of de-identified data from the TriNetX research database. TriNetX contains electronic medical records from nearly 60 large healthcare organizations and contains more than 90 million individual patient records. This proprietary database is available to institutions who contribute to the dataset and can be accessed by researchers at those institutions who adhere to their university policies; thus, it has been well-established for medical research, including but not limited to oral cancer (6), cardiovascular illness (7), and surgical outcomes (8), to name a few. TriNetX, LLC is compliant with the Health Insurance Portability and Accountability Act (HIPAA), the United States federal law which protects the privacy and security of healthcare data, and any additional data privacy regulations applicable to the contributing HCO (9). TriNetX is certified to the ISO 27001:2013 standard and maintains an Information Security Management System (ISMS) to ensure the protection of the healthcare data it has access to and to meet the requirements of the HIPAA Security Rule. Any data displayed on the TriNetX Platform in aggregate form, or any patient level data provided in a data set generated by the TriNetX Platform only contains de-identified data as per the de-identification standard defined in Section §164.514(a) of the HIPAA Privacy Rule (9).

We identified all patients under the age of 25 between November 13, 2002 and November 13, 2022, as the data were extracted on November 15, 2022. From this patient population, we separated initially into two cohorts based on presence or absence of an ADHD diagnosis (ICD: F90). The group with ADHD is the “Overall ADHD” group, and the group without ADHD was labelled the “Neurotypical” group. We then created six subgroups within the ADHD group based on medication regimen, which can be understood as three cohorts of nonexclusive medication use and three cohorts of exclusive medication use: the “no meds” cohort had never taken an FDA-approved ADHD medication (10); the “any meds” cohort had used at least one of the identified medications, and could have used any combination or switched between regimens; the “stimulants” cohort had used at least one of the -amphetamine and -phenidate class medications, and could have used any combination or switched between regimens; the “-phenidate” cohort had only exclusively used medications of the -phenidate class, such as dexmethylphenidate or methylphenidate; the “-etamine” cohort had only exclusively used medications of the -amphetamine class, such as dextroamphetamine or amphetamine; the “non-stimulant” cohort had only exclusively used medications of the FDA-approved non-stimulant ADHD medications, such as atomoxetine or guanfacine (10). Demographic information has been provided in Supplementary Table S1.

The events of interest in this study were identified as follows: “central fractures,” consisting of fractures of the skull, face, cervical vertebrae, spine, ribs, or pelvis (ICD-10: S02, S12, S22, S32); “upper limb fractures,” consisting of fractures of the shoulder, humerus, forearm, wrist, or hand (ICD-10: S42, S52, S62); “lower limb fractures,” consisting of fractures of the femur, lower leg, ankle, or foot (ICD-10: S72, S82, S92); or “any fractures,” consisting of any prior mentioned fracture type.

**Figure 1:**
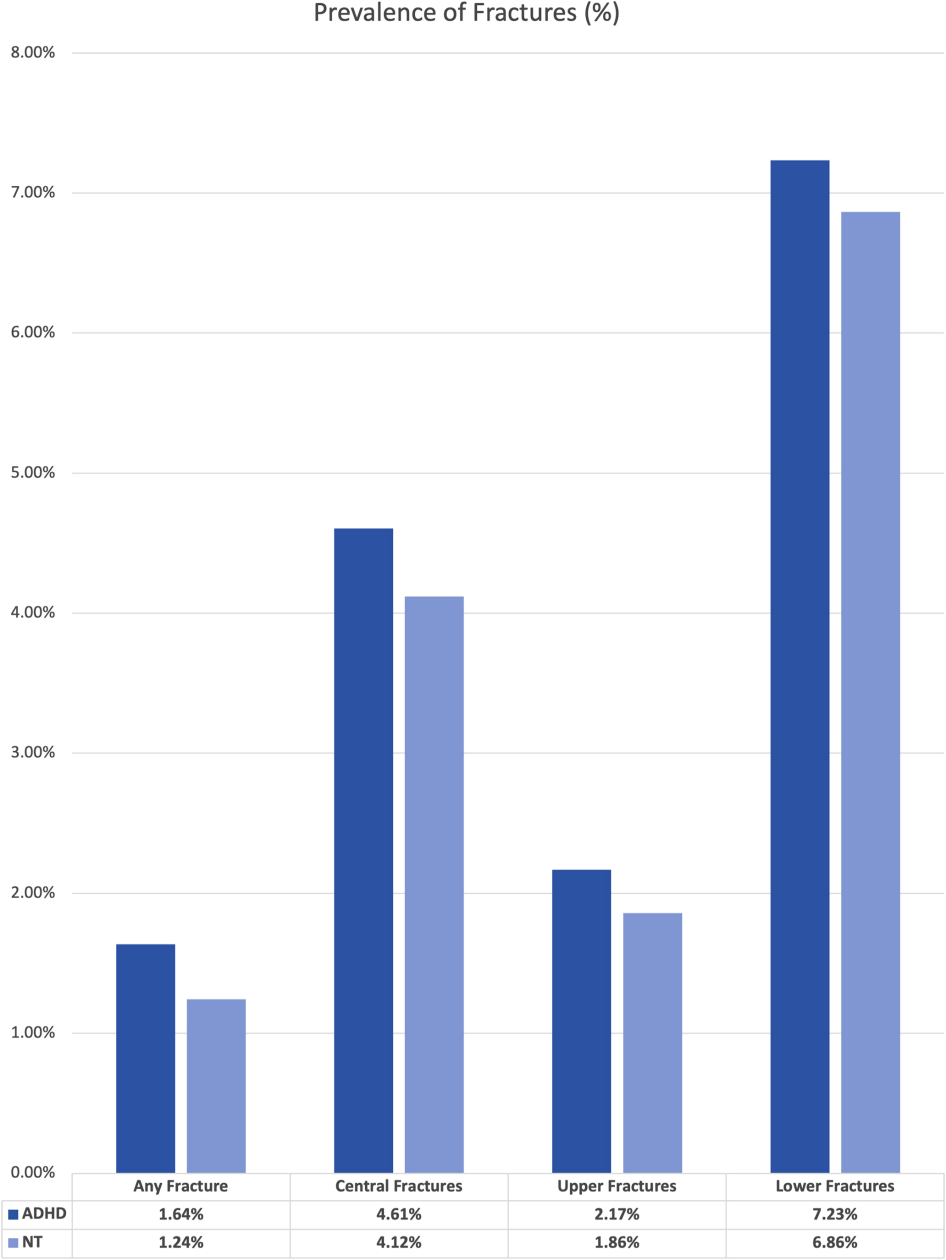
Absolute risk of fracture in overall ADHD and neurotypical cohorts.

We performed two sets of analysis using the TriNetX statistical software. First, we established the overall prevalence of our events by comparing the base rate of each fracture type within the large ADHD group to the “neurotypical” or not ADHD group. The results of this set of analysis are presented in Figure 1. For this analysis, we did not control for any variables within the two cohorts.

Following this analysis, we sought to isolate the effect of ADHD medications on fracture rates. We established the “no medication” ADHD group as the baseline, as they have the neurobiology of ADHD without any medication effects. We then balanced each ADHD subgroup to this baseline cohort based on age, sex, race, and ethnicity using nearest-neighbor matching to a difference between propensity scores <0.1 (9). The identified characteristics utilized in nearest-neighbor matching, as well as pre- and post- matching *t*-test values for these characteristics, are presented for each sub-group in Supplementary Table S1. The outcome of this methodology was that each patient within a medication based sub-group had a peer in the “no meds” cohort who had no significant difference based on age, sex, race, or ethnicity.

**Figure 2:**
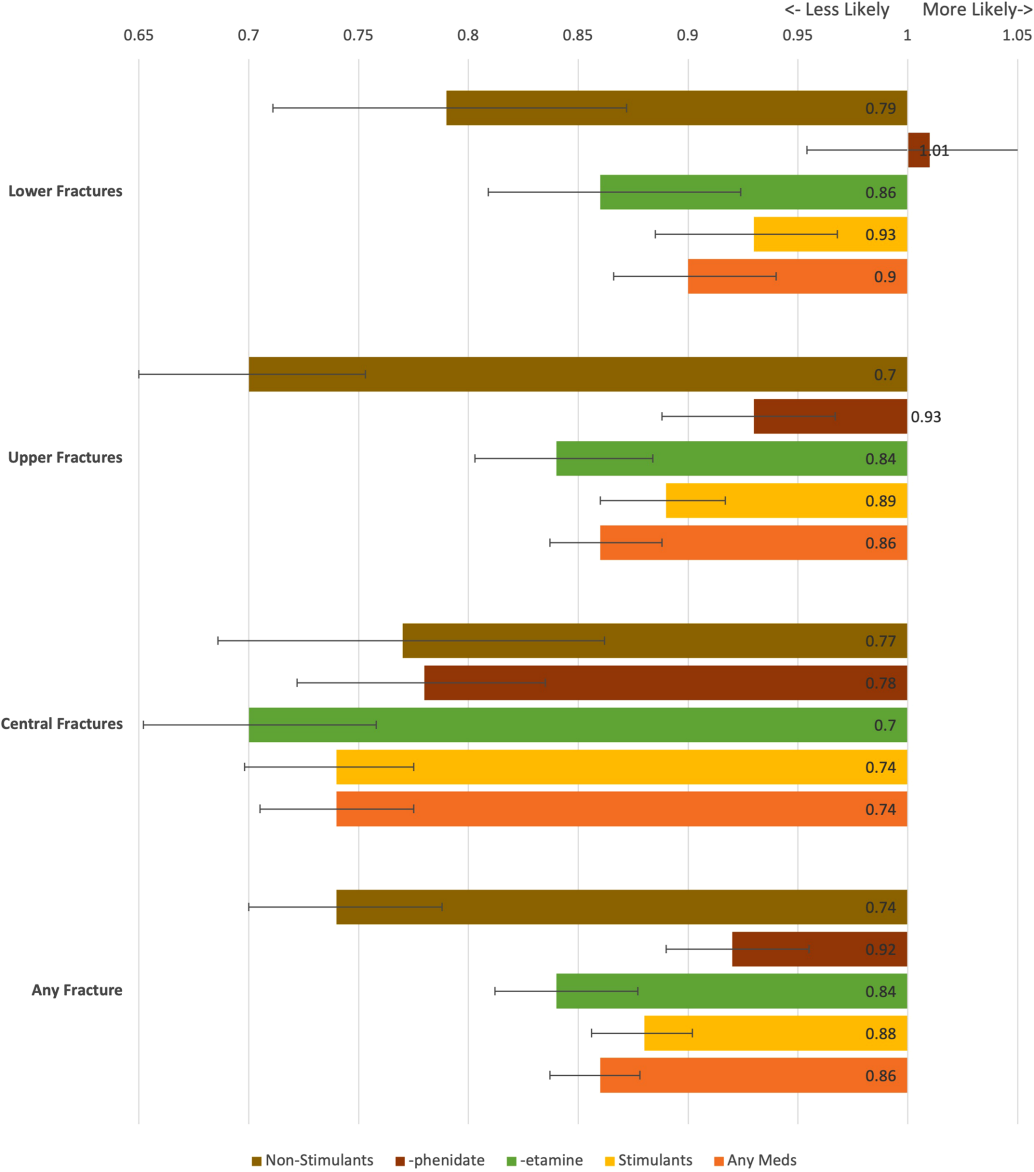
Odds ratio for fractures by medication type. Confidence bars depict a 95% CI. A value greater than 1 indicates the fracture is more likely in ADHD patients on that medication, while a value under 1 indicates the fracture is less likely.

Once the cohorts were balanced, event rates were extracted from the relevant patient records, and odds ratios with 95% confidence intervals were calculated from the given incidence of each event. Risk reduction was calculated as the risk of event in the baseline group minus the risk of event in the comparison group. The baseline event rate for the “no meds” is not provided, as the individuals analyzed varied based on matching to the relevant medication sub-group. The results of this set of analysis are collected in Table 1 and presented graphically in Figure 2. Significance for this study was set at a two-tailed *p*-value <0.05. As this study contained only deidentified aggregate data, the Colorado Multiple Institutional Review Board (COMIRB) designated it as non-human research not in need of approval.

**Table 1:** Cohort size, absolute risk, odds ratio, and *t*test *p*-value by outcome (ICD-10).

Medication Cohort	Cohort N	Event N	Event Risk	Risk Reduction	OR	95% CI
**Any Fracture**						
Any Med	276,953	13,223	4.77%	−0.79%	0.86	(0.837, 0.878)
Stimulant	228,567	11,278	4.93%	−0.67%	0.88	(0.856, 0.902)
-etamine	102,876	5,035	4.89%	−0.90%	0.84	(0.812, 0.877)
-phenidate	125,691	6,243	4.97%	−0.42%	0.92	(0.89, 0.955)
Non-Stimulant	49,801	2,016	4.05%	−1.39%	0.74	(0.7, 0.788)
**Central Fracture**						
Any Med	276,953	2,995	1.08%	−0.79%	0.74	(0.705, 0.775)
Stimulant	228,567	2,491	1.09%	−0.67%	0.74	(0.698, 0.775)
-etamine	102,876	1,179	1.15%	−0.90%	0.7	(0.652, 0.758)
-phenidate	125,691	1,312	1.04%	−0.42%	0.78	(0.722, 0.835)
Non-Stimulant	49,801	528	1.06%	−1.39%	0.77	(0.686, 0.862)
**Upper Fracture**						
Any Med	276,953	8,573	3.10%	−0.79%	0.86	(0.837, 0.888)
Stimulant	228,567	7,368	3.22%	−0.67%	0.89	(0.86, 0.917)
-etamine	102,876	3,224	3.13%	−0.90%	0.84	(0.803, 0.884)
-phenidate	125,691	4,144	3.30%	−0.42%	0.93	(0.888, 0.967)
Non-Stimulant	49,801	1,250	2.51%	−1.39%	0.7	(0.65, 0.753)
**Lower Fracture**						
Any Med	276,953	4,400	1.59%	−0.79%	0.9	(0.866, 0.94)
Stimulant	228,567	3,752	1.64%	−0.67%	0.93	(0.885, 0.968)
-etamine	102,876	1,647	1.60%	−0.90%	0.86	(0.809, 0.924)
-phenidate	125,691	2,105	1.67%	−0.42%	1.01	(0.954, 1.078)
Non-Stimulant	49,801	668	1.34%	−1.39%	0.79	(0.711, 0.872)

## Results

The original size of each cohort is included in Supplementary Table S1. The unmatched analysis between the “neurotypical” or “not ADHD” cohort and the “overall ADHD” cohort revealed higher rates of fractures for all types. The difference in overall prevalence ranged from 0.31% increase for upper limb fractures to 0.49% increase for central fractures.

After matching, the cohort size for each event varied from 668 to 13,223. All but one cohort had a significant difference in each fracture type, with all differences being reduced from the baseline, which was the cohort of ADHD patients without any medication use. Patients in the “phenidate” cohort had an insignificant difference in risk of lower limb fractures. Patients in the “any medication,” “-etamine,” “stimulant,” and “not ADHD” groups all had significant decreased risks for all fracture types; detailed results are in Table 1. Figure 2 presents a visual depiction of odds ratios and confidence intervals between the identified medication type and the matched portion of the “no meds” cohort.

## Discussion

In our uncontrolled analysis between neurotypical individuals and all individuals with ADHD, all fracture outcomes occurred more commonly in the ADHD cohort. This replicates an important observation, and likely contributes to the higher rates of injuries in many aspects of ADHD, including traumatic and athletic injuries (3, 11). Compared to the baseline cohort of patients with ADHD who have never taken one of the identified medications, all medications cohorts had significantly reduced risks across most fracture types. The only exception is for patients in the “-phenidate” cohort, with exclusive use of stimulant medications related to methylphenidate, had increased risk for central, upper limb, and any fracture types, but had an insignificant difference in risk of lower limb fractures.

The neurobiology of ADHD seems to affect nearly all aspects of health (12). While some aspects are obvious and relatively well studied, such as mental health and social functioning (13), recent research has shown ADHD to be more impactful than previously thought. Medication regimen analyses, similar to the one performed in this study, have shown improvements for patients with ADHD on stimulant medication in a wide range of situations from transplant surgery to obstetric complications (14, 15). While an increasing amount of research is being performed on this important issue, much of it compares cohorts with ADHD to neurotypical cohorts, or individuals who likely do not have the neurobiology found in ADHD. In contrast, our study uses a baseline of individuals with ADHD and without medication treatment, which allows us to better isolate the impact of an individual medication regimen.

There are many available treatments for ADHD, and more are approved every year. For young children, the first line treatment is behavioral intervention on the part of the parents, rather than medications (16). In older children or young adults, there are a wide variety of medication options available, which can essentially be separated into stimulants and non-stimulants. Stimulant medications are those designed to act on the dopaminergic pathways in the brain, creating a neurochemical environment that is more like a neurotypical brain (17). Rather than simply addressing behavioral symptoms, stimulant medications target one of the known neurobiological differences present in ADHD. In contrast, non-stimulant medications have a weaker impact on the dopaminergic pathways and are used commonly for symptomatic management when stimulants are not appropriate (18).

Many patients, parents, and providers are concerned over the potential side effects of medication types. Within stimulants, methylphenidate is known to decrease the bone density with chronic use in both animal models and human studies (19, 20). This has been proposed as a potential cause for the increased absolute risk of fractures for patients with ADHD compared to neurotypical individuals (21). Interestingly, a large human study of chronic methylphenidate treatment has shown a decreased risk of fracture for individuals on ADHD compared to individuals with ADHD who are not on treatment (22). Our data presents a similar trend. While there is an increased absolute risk of fractures in all patients with ADHD compared to neurotypical individuals, our data showed a decreased risk for 3 out of 4 studied types of fractures in the “-phenidate” group compared to patients with ADHD who do not use medication, and an insignificant difference in lower limb fractures. If methylphenidate decreased bone density to a clinically significant extent, we would likely expect an increase compared to individuals with ADHD who have not been treated with any medication and compared to individuals who had been treated with amphetamine-type stimulants. Instead, our data suggests that bone loss due to methylphenidate is not a clinically significant factor in the increased fracture risk for patients with ADHD and confirms prior research that methylphenidate use may be protective against fractures (21).

While both stimulants and non-stimulants are known to impact behavior, stimulant medications have more frequently been the focus of research. Behavioral changes associated with stimulant medications are known to decrease the chance of fracture in patients with ADHD (21). Our data reinforces these findings, and further suggests that non-stimulants may additionally reduce the risk of fractures through a similar behavioral mechanism. It is also notable that within our study, non-stimulants had the greatest difference in absolute risk reduction of all medication types, although the confidence intervals overlapped with stimulant regimen for upper and central fractures. Given the overlapping confidence intervals, there is no statistically significant difference by treatment regimens for central fractures, potentially due to the relatively small sample size compared to other types of fractures. As some non-stimulants are also used as sedatives (18), one possibility for the difference in fractures could be a reduction in baseline activity. However, it is worth noting that non-stimulant medications are most often used for individuals with less severe symptomatology (23). An individual with less severe symptoms may have a lower fracture risk at baseline, and it is unknown how much of the effect seen in our study is related to the medication as opposed to baseline differences. As non-stimulants are not considered to contribute to loss of bone density (24), this study adds to the knowledge of the field by deepening the available evidence that compares medication types.

Our study is not without limitations. As with all studies involving deidentified, aggregate data, we were unable to trace individuals throughout time or multiple simultaneous events; for example, a patient who fractured their skull, humerus, and femur in the same incident would be present once in each event analysis, while someone who fractured their humerus and repeatedly presented to medical care for pain medication would be present in the data one time for each visit. Furthermore, we did not separate out different types of fracture such as pathological, stress, or traumatic, which may have presented different data. Additionally, we did not directly compare amphetamines and methylphenidates, although substantial differences between amphetamines and methylphenidate medications are recognized in the literature, with amphetamines often having longer effects (25). While a causal analysis is not possible in our study, our study provides a basis for further investigation and comparison between amphetamine and methylphenidate medications, with amphetamines potentially being more effective for fracture prevention. Finally, we were limited by the available data and were unable to control for severity of ADHD symptoms, although some information can be presumed based on the type of medication used.

This study offers new information that may aid providers in providing information to patients and families regarding the impact of ADHD treatment on bone health. As patients experiment with different medication regimens, providers should be aware of the difference in risk of bone fracture by medication type. Furthermore, our manuscript is the first to directly compare fracture risks in non-stimulant and stimulant medications, finding intriguing differences across medication types that cannot be understood at a causal level from the available data. However, by highlighting potential differences in fractures across medication classes, our results highlight the need for continued research to better discern appropriate medication regimens for individual patients with the goal of improving overall risk reduction and producing better outcomes for individuals with ADHD.

## Data Availability

The original contributions presented in the study are included in the article/**Supplementary Materials**, further inquiries can be directed to the corresponding author.’
