# Supplementary material for: Fracture rates by medication type in attention-deficit/hyperactive disorder
Source: Front Surg. 2023 Feb 15;10:973266. doi: 10.3389/fsurg.2023.973266 (PMC9975348; doi:10.3389/fsurg.2023.973266)
Supplement: Supplementary file 2 [file Datasheet1.docx]

| **Cohort 1 and cohort 2 patient count before and after propensity score matching** | | | | | | | | | | | |
| --- | --- | --- | --- | --- | --- | --- | --- | --- | --- | --- | --- |
|  | | | Cohort | | | Patient count before matching | | | Patient count after matching | | |
|  | | | 1 – Stimulant Medications <25 | | | 231,185 | | | 231,162 | | |
|  | | | 2 - No Meds <25 | | | 354,429 | | | 231,162 | | |
| **Cohort 1 (N = 231,185) and cohort 2 (N = 354,429) characteristics before propensity score matching** | | | | | | | | | | | |
|  | **Demographics** | | | | | | | | | | |
|  |  | Cohort | | |  | Mean ± SD | Patients | % of Cohort | | P-Value | Std diff. |
|  |  | 1 2 | | Age | Current Age | 17.0 +/- 5.0 16.4 +/- 5.5 | 231,162 353,961 | 100% 100% | | <0.001 | 0.127 |
|  |  | 1 2 | | 2106-3 | White |  | 149,455 206,418 | 64.7% 58.3% | | <0.001 | 0.131 |
|  |  | 1 2 | | F | Female |  | 80,488 120,294 | 34.8% 34.0% | | <0.001 | 0.018 |
|  |  | 1 2 | | UN | Unknown Ethnicity |  | 46,724 94,221 | 20.2% 26.6% | | <0.001 | 0.152 |
|  |  | 1 2 | | 2054-5 | Black or African American |  | 39,589 65,771 | 17.1% 18.6% | | <0.001 | 0.038 |
|  |  | 1 2 | | 2135-2 | Hispanic or Latino |  | 22,434 38,899 | 9.7% 11.0% | | <0.001 | 0.042 |
|  |  | 1 2 | | 2186-5 | Not Hispanic or Latino |  | 162,004 220,841 | 70.1% 62.4% | | <0.001 | 0.163 |
|  |  | 1 2 | | M | Male |  | 150,601 233,548 | 65.1% 66.0% | | <0.001 | 0.018 |
|  |  | 1 2 | | 2131-1 | Unknown Race |  | 38,441 74,546 | 16.6% 21.1% | | <0.001 | 0.113 |
|  |  | 1 2 | | 2028-9 | Asian |  | 2,532 5,320 | 1.1% 1.5% | | <0.001 | 0.036 |
| **Cohort 1 (N = 231,162) and cohort 2 (N = 231,162) characteristics after propensity score matching** | | | | | | | | | | | |
|  | **Demographics** | | | | | | | | | | |
|  |  | Cohort | | |  | Mean ± SD | Patients | % of Cohort | | P-Value | Std diff. |
|  |  | 1 2 | | Age | Current Age | 17.0 +/- 5.0 17.0 +/- 5.0 | 231,162 231,162 | 100% 100% | | 0.968 | <0.001 |
|  |  | 1 2 | | 2106-3 | White |  | 149,455 149,467 | 64.7% 64.7% | | 0.971 | <0.001 |
|  |  | 1 2 | | F | Female |  | 80,488 80,485 | 34.8% 34.8% | | 0.993 | <0.001 |
|  |  | 1 2 | | UN | Unknown Ethnicity |  | 46,724 46,735 | 20.2% 20.2% | | 0.968 | <0.001 |
|  |  | 1 2 | | 2054-5 | Black or African American |  | 39,589 39,595 | 17.1% 17.1% | | 0.981 | <0.001 |
|  |  | 1 2 | | 2135-2 | Hispanic or Latino |  | 22,434 22,430 | 9.7% 9.7% | | 0.984 | <0.001 |
|  |  | 1 2 | | 2186-5 | Not Hispanic or Latino |  | 162,004 161,997 | 70.1% 70.1% | | 0.982 | <0.001 |
|  |  | 1 2 | | M | Male |  | 150,601 150,626 | 65.1% 65.2% | | 0.938 | <0.001 |
|  |  | 1 2 | | 2131-1 | Unknown Race |  | 38,441 38,478 | 16.6% 16.6% | | 0.884 | <0.001 |
|  |  | 1 2 | | 2028-9 | Asian |  | 2,532 2,510 | 1.1% 1.1% | | 0.755 | 0.001 |

| **Cohort 1 and cohort 2 patient count before and after propensity score matching** | | | | | | | | | | | |
| --- | --- | --- | --- | --- | --- | --- | --- | --- | --- | --- | --- |
|  | | | Cohort | | | Patient count before matching | | | Patient count after matching | | |
|  | | | 1 - <25 -phenidate | | | 127,038 | | | 127,025 | | |
|  | | | 2 - No Meds <25 | | | 354,429 | | | 127,025 | | |
| **Cohort 1 (N = 127,038) and cohort 2 (N = 354,429) characteristics before propensity score matching** | | | | | | | | | | | |
|  | **Demographics** | | | | | | | | | | |
|  |  | Cohort | | |  | Mean ± SD | Patients | % of Cohort | | P-Value | Std diff. |
|  |  | 1 2 | | Age | Current Age | 16.1 +/- 4.9 16.4 +/- 5.5 | 127,025 353,961 | 100% 100% | | <0.001 | 0.057 |
|  |  | 1 2 | | 2106-3 | White |  | 80,402 206,418 | 63.3% 58.3% | | <0.001 | 0.102 |
|  |  | 1 2 | | F | Female |  | 41,212 120,294 | 32.4% 34.0% | | <0.001 | 0.033 |
|  |  | 1 2 | | UN | Unknown Ethnicity |  | 24,610 94,221 | 19.4% 26.6% | | <0.001 | 0.173 |
|  |  | 1 2 | | 2054-5 | Black or African American |  | 22,100 65,771 | 17.4% 18.6% | | <0.001 | 0.031 |
|  |  | 1 2 | | 2135-2 | Hispanic or Latino |  | 13,661 38,899 | 10.8% 11.0% | | 0.021 | 0.008 |
|  |  | 1 2 | | 2186-5 | Not Hispanic or Latino |  | 88,754 220,841 | 69.9% 62.4% | | <0.001 | 0.159 |
|  |  | 1 2 | | M | Male |  | 85,772 233,548 | 67.5% 66.0% | | <0.001 | 0.033 |
|  |  | 1 2 | | 2131-1 | Unknown Race |  | 22,469 74,546 | 17.7% 21.1% | | <0.001 | 0.085 |
|  |  | 1 2 | | 2028-9 | Asian |  | 1,429 5,320 | 1.1% 1.5% | | <0.001 | 0.033 |
| **Cohort 1 (N = 127,025) and cohort 2 (N = 127,025) characteristics after propensity score matching** | | | | | | | | | | | |
|  | **Demographics** | | | | | | | | | | |
|  |  | Cohort | | |  | Mean ± SD | Patients | % of Cohort | | P-Value | Std diff. |
|  |  | 1 2 | | Age | Current Age | 16.1 +/- 4.9 16.1 +/- 4.9 | 127,025 127,025 | 100% 100% | | 0.974 | <0.001 |
|  |  | 1 2 | | 2106-3 | White |  | 80,402 80,399 | 63.3% 63.3% | | 0.990 | <0.001 |
|  |  | 1 2 | | F | Female |  | 41,212 41,215 | 32.4% 32.4% | | 0.990 | <0.001 |
|  |  | 1 2 | | UN | Unknown Ethnicity |  | 24,610 24,604 | 19.4% 19.4% | | 0.976 | <0.001 |
|  |  | 1 2 | | 2054-5 | Black or African American |  | 22,100 22,100 | 17.4% 17.4% | | 1 | <0.001 |
|  |  | 1 2 | | 2135-2 | Hispanic or Latino |  | 13,661 13,662 | 10.8% 10.8% | | 0.995 | <0.001 |
|  |  | 1 2 | | 2186-5 | Not Hispanic or Latino |  | 88,754 88,759 | 69.9% 69.9% | | 0.983 | <0.001 |
|  |  | 1 2 | | M | Male |  | 85,772 85,779 | 67.5% 67.5% | | 0.976 | <0.001 |
|  |  | 1 2 | | 2131-1 | Unknown Race |  | 22,469 22,476 | 17.7% 17.7% | | 0.971 | <0.001 |
|  |  | 1 2 | | 2028-9 | Asian |  | 1,429 1,425 | 1.1% 1.1% | | 0.940 | <0.001 |

| **Cohort 1 and cohort 2 patient count before and after propensity score matching** | | | | | | | | | | | |
| --- | --- | --- | --- | --- | --- | --- | --- | --- | --- | --- | --- |
|  | | | Cohort | | | Patient count before matching | | | Patient count after matching | | |
|  | | | 1 – Non-Stimulants <25 | | | 50,642 | | | 50,636 | | |
|  | | | 2 - No Meds <25 | | | 354,429 | | | 50,636 | | |
| **Cohort 1 (N = 50,642) and cohort 2 (N = 354,429) characteristics before propensity score matching** | | | | | | | | | | | |
|  | **Demographics** | | | | | | | | | | |
|  |  | Cohort | | |  | Mean ± SD | Patients | % of Cohort | | P-Value | Std diff. |
|  |  | 1 2 | | Age | Current Age | 16.4 +/- 5.4 16.4 +/- 5.5 | 50,636 353,961 | 100% 100% | | 0.323 | 0.005 |
|  |  | 1 2 | | 2106-3 | White |  | 33,604 206,418 | 66.4% 58.3% | | <0.001 | 0.167 |
|  |  | 1 2 | | F | Female |  | 18,101 120,294 | 35.7% 34.0% | | <0.001 | 0.037 |
|  |  | 1 2 | | UN | Unknown Ethnicity |  | 9,608 94,221 | 19.0% 26.6% | | <0.001 | 0.183 |
|  |  | 1 2 | | 2054-5 | Black or African American |  | 7,461 65,771 | 14.7% 18.6% | | <0.001 | 0.103 |
|  |  | 1 2 | | 2135-2 | Hispanic or Latino |  | 4,694 38,899 | 9.3% 11.0% | | <0.001 | 0.057 |
|  |  | 1 2 | | 2186-5 | Not Hispanic or Latino |  | 36,334 220,841 | 71.8% 62.4% | | <0.001 | 0.200 |
|  |  | 1 2 | | M | Male |  | 32,522 233,548 | 64.2% 66.0% | | <0.001 | 0.037 |
|  |  | 1 2 | | 2131-1 | Unknown Race |  | 8,762 74,546 | 17.3% 21.1% | | <0.001 | 0.096 |
|  |  | 1 2 | | 2028-9 | Asian |  | 535 5,320 | 1.1% 1.5% | | <0.001 | 0.040 |
| **Cohort 1 (N = 50,636) and cohort 2 (N = 50,636) characteristics after propensity score matching** | | | | | | | | | | | |
|  | **Demographics** | | | | | | | | | | |
|  |  | Cohort | | |  | Mean ± SD | Patients | % of Cohort | | P-Value | Std diff. |
|  |  | 1 2 | | Age | Current Age | 16.4 +/- 5.4 16.4 +/- 5.4 | 50,636 50,636 | 100% 100% | | 0.999 | <0.001 |
|  |  | 1 2 | | 2106-3 | White |  | 33,604 33,605 | 66.4% 66.4% | | 0.995 | <0.001 |
|  |  | 1 2 | | F | Female |  | 18,101 18,102 | 35.7% 35.7% | | 0.995 | <0.001 |
|  |  | 1 2 | | UN | Unknown Ethnicity |  | 9,608 9,608 | 19.0% 19.0% | | 1 | <0.001 |
|  |  | 1 2 | | 2054-5 | Black or African American |  | 7,461 7,461 | 14.7% 14.7% | | 1 | <0.001 |
|  |  | 1 2 | | 2135-2 | Hispanic or Latino |  | 4,694 4,694 | 9.3% 9.3% | | 1 | <0.001 |
|  |  | 1 2 | | 2186-5 | Not Hispanic or Latino |  | 36,334 36,334 | 71.8% 71.8% | | 1 | <0.001 |
|  |  | 1 2 | | M | Male |  | 32,522 32,522 | 64.2% 64.2% | | 1 | <0.001 |
|  |  | 1 2 | | 2131-1 | Unknown Race |  | 8,762 8,787 | 17.3% 17.4% | | 0.836 | 0.001 |
|  |  | 1 2 | | 2028-9 | Asian |  | 535 535 | 1.1% 1.1% | | 1 | <0.001 |

| **Cohort 1 and cohort 2 patient count before and after propensity score matching** | | | | | | | | | | | |
| --- | --- | --- | --- | --- | --- | --- | --- | --- | --- | --- | --- |
|  | | | Cohort | | | Patient count before matching | | | Patient count after matching | | |
|  | | | 1 - <25 -etamine | | | 104,147 | | | 104,137 | | |
|  | | | 2 - No Meds <25 | | | 354,429 | | | 104,137 | | |
| **Cohort 1 (N = 104,147) and cohort 2 (N = 354,429) characteristics before propensity score matching** | | | | | | | | | | | |
|  | **Demographics** | | | | | | | | | | |
|  |  | Cohort | | |  | Mean ± SD | Patients | % of Cohort | | P-Value | Std diff. |
|  |  | 1 2 | | Age | Current Age | 18.2 +/- 4.8 16.4 +/- 5.5 | 104,137 353,961 | 100% 100% | | <0.001 | 0.357 |
|  |  | 1 2 | | 2106-3 | White |  | 69,053 206,418 | 66.3% 58.3% | | <0.001 | 0.166 |
|  |  | 1 2 | | F | Female |  | 39,276 120,294 | 37.7% 34.0% | | <0.001 | 0.078 |
|  |  | 1 2 | | UN | Unknown Ethnicity |  | 22,114 94,221 | 21.2% 26.6% | | <0.001 | 0.126 |
|  |  | 1 2 | | 2054-5 | Black or African American |  | 17,489 65,771 | 16.8% 18.6% | | <0.001 | 0.047 |
|  |  | 1 2 | | 2135-2 | Hispanic or Latino |  | 8,773 38,899 | 8.4% 11.0% | | <0.001 | 0.087 |
|  |  | 1 2 | | 2186-5 | Not Hispanic or Latino |  | 73,250 220,841 | 70.3% 62.4% | | <0.001 | 0.169 |
|  |  | 1 2 | | M | Male |  | 64,829 233,548 | 62.3% 66.0% | | <0.001 | 0.078 |
|  |  | 1 2 | | 2131-1 | Unknown Race |  | 15,972 74,546 | 15.3% 21.1% | | <0.001 | 0.149 |
|  |  | 1 2 | | 2028-9 | Asian |  | 1,103 5,320 | 1.1% 1.5% | | <0.001 | 0.039 |
| **Cohort 1 (N = 104,137) and cohort 2 (N = 104,137) characteristics after propensity score matching** | | | | | | | | | | | |
|  | **Demographics** | | | | | | | | | | |
|  |  | Cohort | | |  | Mean ± SD | Patients | % of Cohort | | P-Value | Std diff. |
|  |  | 1 2 | | Age | Current Age | 18.2 +/- 4.8 18.2 +/- 4.8 | 104,137 104,137 | 100% 100% | | 0.997 | <0.001 |
|  |  | 1 2 | | 2106-3 | White |  | 69,053 69,053 | 66.3% 66.3% | | 1 | <0.001 |
|  |  | 1 2 | | F | Female |  | 39,276 39,281 | 37.7% 37.7% | | 0.982 | <0.001 |
|  |  | 1 2 | | UN | Unknown Ethnicity |  | 22,114 22,112 | 21.2% 21.2% | | 0.991 | <0.001 |
|  |  | 1 2 | | 2054-5 | Black or African American |  | 17,489 17,490 | 16.8% 16.8% | | 0.995 | <0.001 |
|  |  | 1 2 | | 2135-2 | Hispanic or Latino |  | 8,773 8,772 | 8.4% 8.4% | | 0.994 | <0.001 |
|  |  | 1 2 | | 2186-5 | Not Hispanic or Latino |  | 73,250 73,253 | 70.3% 70.3% | | 0.989 | <0.001 |
|  |  | 1 2 | | M | Male |  | 64,829 64,831 | 62.3% 62.3% | | 0.993 | <0.001 |
|  |  | 1 2 | | 2131-1 | Unknown Race |  | 15,972 16,021 | 15.3% 15.4% | | 0.766 | 0.001 |
|  |  | 1 2 | | 2028-9 | Asian |  | 1,103 1,102 | 1.1% 1.1% | | 0.983 | <0.001 |

| **Cohort 1 and cohort 2 patient count before and after propensity score matching** | | | | | | | | | | | |
| --- | --- | --- | --- | --- | --- | --- | --- | --- | --- | --- | --- |
|  | | | Cohort | | | Patient count before matching | | | Patient count after matching | | |
|  | | | 1 - <25 Any individual Med | | | 281,827 | | | 280,367 | | |
|  | | | 2 - No Meds <25 | | | 354,429 | | | 280,367 | | |
| **Cohort 1 (N = 281,827) and cohort 2 (N = 354,429) characteristics before propensity score matching** | | | | | | | | | | | |
|  | **Demographics** | | | | | | | | | | |
|  |  | Cohort | | |  | Mean ± SD | Patients | % of Cohort | | P-Value | Std diff. |
|  |  | 1 2 | | Age | Current Age | 16.9 +/- 5.0 16.4 +/- 5.5 | 281,798 353,961 | 100% 100% | | <0.001 | 0.105 |
|  |  | 1 2 | | 2106-3 | White |  | 183,059 206,418 | 65.0% 58.3% | | <0.001 | 0.137 |
|  |  | 1 2 | | F | Female |  | 98,589 120,294 | 35.0% 34.0% | | <0.001 | 0.021 |
|  |  | 1 2 | | UN | Unknown Ethnicity |  | 56,332 94,221 | 20.0% 26.6% | | <0.001 | 0.157 |
|  |  | 1 2 | | 2054-5 | Black or African American |  | 47,050 65,771 | 16.7% 18.6% | | <0.001 | 0.049 |
|  |  | 1 2 | | 2135-2 | Hispanic or Latino |  | 27,128 38,899 | 9.6% 11.0% | | <0.001 | 0.045 |
|  |  | 1 2 | | 2186-5 | Not Hispanic or Latino |  | 198,338 220,841 | 70.4% 62.4% | | <0.001 | 0.170 |
|  |  | 1 2 | | M | Male |  | 183,123 233,548 | 65.0% 66.0% | | <0.001 | 0.021 |
|  |  | 1 2 | | 2131-1 | Unknown Race |  | 47,203 74,546 | 16.8% 21.1% | | <0.001 | 0.110 |
|  |  | 1 2 | | 2028-9 | Asian |  | 3,067 5,320 | 1.1% 1.5% | | <0.001 | 0.037 |
| **Cohort 1 (N = 280,367) and cohort 2 (N = 280,367) characteristics after propensity score matching** | | | | | | | | | | | |
|  | **Demographics** | | | | | | | | | | |
|  |  | Cohort | | |  | Mean ± SD | Patients | % of Cohort | | P-Value | Std diff. |
|  |  | 1 2 | | Age | Current Age | 16.9 +/- 5.0 16.9 +/- 5.0 | 280,367 280,367 | 100% 100% | | 0.012 | 0.007 |
|  |  | 1 2 | | 2106-3 | White |  | 181,641 180,828 | 64.8% 64.5% | | 0.023 | 0.006 |
|  |  | 1 2 | | F | Female |  | 97,803 96,871 | 34.9% 34.6% | | 0.009 | 0.007 |
|  |  | 1 2 | | UN | Unknown Ethnicity |  | 56,332 56,340 | 20.1% 20.1% | | 0.979 | <0.001 |
|  |  | 1 2 | | 2054-5 | Black or African American |  | 47,050 47,828 | 16.8% 17.1% | | 0.006 | 0.007 |
|  |  | 1 2 | | 2135-2 | Hispanic or Latino |  | 27,128 27,231 | 9.7% 9.7% | | 0.642 | 0.001 |
|  |  | 1 2 | | 2186-5 | Not Hispanic or Latino |  | 196,907 196,796 | 70.2% 70.2% | | 0.746 | 0.001 |
|  |  | 1 2 | | M | Male |  | 182,481 183,417 | 65.1% 65.4% | | 0.009 | 0.007 |
|  |  | 1 2 | | 2131-1 | Unknown Race |  | 47,190 47,405 | 16.8% 16.9% | | 0.443 | 0.002 |
|  |  | 1 2 | | 2028-9 | Asian |  | 3,067 2,942 | 1.1% 1.0% | | 0.105 | 0.004 |

| **Cohort 1 and cohort 2 patient count** | | | | | | | | | | | |
| --- | --- | --- | --- | --- | --- | --- | --- | --- | --- | --- | --- |
|  | | | Cohort | | | Patient count | | |  | | |
|  | | | 1 - <25 Overall ADHD | | | 870,159 | | |  | | |
|  | | | 2 - <25 Neurotypical | | | 19,299,664 | | |  | | |
| **Cohort 1 (N = 870,159) and cohort 2 (N = 19,299,664) characteristics** | | | | | | | | | | | |
|  | **Demographics** | | | | | | | | | | |
|  |  | Cohort | | |  | Mean ± SD | Patients | % of Cohort | | P-Value | Std diff. |
|  |  | 1 2 | | Age | Current Age | 16.5 +/- 5.2 14.0 +/- 7.1 | 869,284 18,981,056 | 100% 100% | | <0.001 | 0.400 |
|  |  | 1 2 | | 2106-3 | White |  | 546,981 9,607,911 | 62.9% 50.6% | | <0.001 | 0.250 |
|  |  | 1 2 | | F | Female |  | 293,201 9,538,177 | 33.7% 50.3% | | <0.001 | 0.340 |
|  |  | 1 2 | | UN | Unknown Ethnicity |  | 187,910 6,531,424 | 21.6% 34.4% | | <0.001 | 0.288 |
|  |  | 1 2 | | 2186-5 | Not Hispanic or Latino |  | 594,683 9,580,507 | 68.4% 50.5% | | <0.001 | 0.372 |
|  |  | 1 2 | | 2135-2 | Hispanic or Latino |  | 86,691 2,869,125 | 10.0% 15.1% | | <0.001 | 0.156 |
|  |  | 1 2 | | 2054-5 | Black or African American |  | 151,639 3,039,084 | 17.4% 16.0% | | <0.001 | 0.038 |
|  |  | 1 2 | | M | Male |  | 575,795 9,411,618 | 66.2% 49.6% | | <0.001 | 0.342 |
|  |  | 1 2 | | 2131-1 | Unknown Race |  | 155,818 5,625,845 | 17.9% 29.6% | | <0.001 | 0.278 |
|  |  | 1 2 | | 2028-9 | Asian |  | 10,334 569,796 | 1.2% 3.0% | | <0.001 | 0.127 |
